# Effects of beef fat enriched with *trans* vaccenic acid and *cis*9, *trans*11-CLA on glucose homoeostasis and hepatic lipid accumulation in high-fat diet-induced obese mice

**DOI:** 10.1017/S000711452400062X

**Published:** 2024-06-28

**Authors:** Yanqing Xu, Ming-Fo Hsu, Fawaz George Haj, Payam Vahmani

**Affiliations:** 1 Department of Animal Science, University of California Davis, One Shields Ave, Davis, CA 95616, USA; 2 Department of Nutrition, University of California Davis, One Shields Ave, Davis, CA 95616, USA

**Keywords:** Beef, Biohydrogenation, *trans*-FA, Type 2 diabetes

## Abstract

*Trans* vaccenic acid (TVA, *trans*11–18 : 1) and *cis*9, *trans*11-CLA (also known as rumenic acid; RA) have received widespread attention as potentially beneficial *trans*-FA due to their putative health benefits, including anti-diabetic properties. The objective of this study was to determine the effects of beef fat naturally enriched with TVA and RA on parameters related to glucose homoeostasis and associated metabolic markers in diet-induced obese (DIO) mice. Thirty-six male C57BL/6J mice (8 weeks old) were fed for 19 weeks with either a control low-fat diet (CLF), a control high-fat diet (CHF), or a TVA+RA-enriched high-fat diet (EHF). Compared with CLF, feeding either CHF or EHF resulted in adverse metabolic outcomes associated with high-fat diets, including adiposity, impaired glucose control and hepatic steatosis. However, the EHF diet induced a significantly higher liver weight TAG content and elevated plasma alanine transaminase levels compared with the CHF diet. Collectively, the findings from this study suggest that EHF does not improve glucose tolerance and worsens liver steatosis in DIO mice. However, the adverse effects of EHF on the liver could be in part related to the presence of other *trans*-FA in the enriched beef fat.

Milk and meat fats from ruminant animals (e.g. cattle, sheep and goats) have the most complex fatty acid composition (>100 different fatty acids) among all edible fats, in part due to the biohydrogenation process^(1)^. In ruminants, dietary unsaturated fatty acids are toxic to rumen bacteria. To cope, rumen bacteria convert them to less toxic saturated fatty acids through biohydrogenation. During this process, numerous biohydrogenation intermediates are produced, and a portion of them pass from the rumen and subsequently find their way into tissues and milk after post-ruminal absorption. Given that the majority of these intermediates contain at least one *trans* double bond, they are generally referred to as ‘ruminant’ or ‘natural’ *trans*-FA (TFA). There are at least forty different TFA isomers found in ruminant-derived fats, with *trans* vaccenic acid (TVA; *t*11–18 : 1) and *cis(c)*9, *trans(t)*11-CLA (*c*9,*t*11-CLA; also known as rumenic acid, RA) being the most predominant ones, accounting for 50–70 % of total TFA in ruminant-derived fats^(2)^. TVA can also be converted to RA in the body via Δ-9 desaturation, with the conversion rate estimated to be approximately 19 % in humans^(2)^.

In contrast to partially hydrogenated vegetable oils (also known as ‘industrial’ TFA), which have undisputable adverse health effects, particularly increased CVD risk and mortality, TVA and RA have been associated with reduced risk of some disease conditions, including type 2 diabetes^(3–7)^. In rodent studies, supplementation with pure TVA or RA reduces fasting and postprandial insulin levels and homeostatic model assessment for insulin resistance^(8–11)^. The apparent insulin-sensitising effects of TVA and RA in these studies were mainly attributed to their potential to bind and activate peroxisome proliferator-activated receptor gamma-regulated pathways in the liver and adipose tissues^(10–12)^. Moreover, TVA has been shown to restore glucose homoeostasis in diabetic rats by promoting insulin secretion from pancreatic islets^(13)^.

Given the postulated health benefits of TVA and RA, ruminant nutritionists have sought to develop feeding strategies to enhance the content of these fatty acids in beef and dairy products^(14)^. The findings from these studies have shown that significant enrichment with TVA and RA can be achieved by feeding cattle forage-based diets that are supplemented with PUFA sources, such as oilseeds (e.g. flaxseed or sunflower seeds)^(14)^.

A limited number of rodent studies have shown that feeding butter from oilseed-feed dairy cattle (i.e. TVA + RA-enriched butter) improved plasma lipoprotein profiles compared with regular butter^(15–18)^. Furthermore, feeding TVA + RA-enriched beef or milk fats alleviated insulin resistance and glucose intolerance in obese/insulin-resistant JCR:LA-cp rats and high-fat-fed Wistar rats, respectively, when compared with those fed regular beef or milk fats^(11,19)^. However, the effects of feeding TVA + RA-enriched ruminant fats on glucose homoeostasis have not been studied in diet-induced obese (DIO) mice, a clinically translatable animal model, to test the efficacy of natural compounds and/or drugs against prediabetes and type 2 diabetes. Thus, we sought to determine whether long-term supplementation with TVA + RA-enriched beef fat would improve glucose homoeostasis and associated metabolic markers in DIO mice. We hypothesised that dietary supplementation with TVA + RA-enriched beef fat would attenuate glucose intolerance, insulin resistance and other obesity-associated metabolic impairments in DIO mice.

## Experimental methods

### Animals and diets

All animal protocols were approved by the Institutional Animal Care and Use Committee of the University of California, Davis (protocol #23582) and carried out in accordance with the ARRIVE guidelines and the National Institutes of Health Guide for the Care and Use of Laboratory Animals.

A total of thirty-six 8-week-old C57BL/6J male mice were obtained from Jackson Laboratories (Bar Harbor). The mice were group-housed (four mice/cage) and kept under a 12 h light cycle (07.00. lights on, 19.00 lights off) in a temperature (22°C) and humidity-controlled vivarium with *ad libitum* access to food and water. After 1 week of acclimation to a standard rodent-chow diet, each cage was randomly assigned to either a control low-fat diet (10 % energy from fat), a control high-fat diet (CHF, 45 % energy from fat) with no TFA or an enriched high-fat diet (EHF, 45 % energy from fat) containing beef tallow enriched with TVA and RA (Tables 1 and 2). For CHF, we used lard (i.e. a non-ruminant fat source) to create a TFA-free high-fat diet. Furthermore, we matched the TFA (*trans*18 : 1, *cis,trans*18 : 2, *cis,trans,cis*18 : 3) in EHF with their *cis* equivalents (*cis*18 : 1, *cis,cis*18 : 2, *cis,cis,cis*18 : 3) in CHF by including different plant-based oils (cocoa butter, safflower oil and soyabean oil; Table 2). As a result, CHF and EHF had a similar content of SFA, MUFA, PUFA and a similar *n*-3/*n*-6 PUFA ratio (Table 3).

**Table 1.** Diet formulations

	Control low-fat (CLF)		Control high-fat (CHF)		Enriched high-fat (EHF)	
Ingredients	g/kg	kcal%	g/kg	kcal%	g/kg	kcal%
Casein, 30 mesh	189·56	19·72	233·06	19·72	233·06	19·72
l-Cystine	2·84	0·30	3·50	0·30	3·50	0·30
Corn starch	428·61	44·58	84·83	7·17	84·83	7·17
Maltodextrin 10	71·09	7·39	116·53	9·86	116·53	9·86
Sucrose	163·78	17·04	201·36	17·03	201·36	17·03
Cellulose, BW200	47·39	0·00	58·26	0·00	58·26	0·00
Soyabean oil	23·70	5·55	28·31	5·39	0·00	0·00
Lard	18·96	4·44	179·33	34·15	0·00	0·00
Flax-fed beef tallow	0·00	0·00	0·00	0·00	169·89	32·35
Safflower oil	0·00	0·00	11·80	2·25	30·68	5·84
Cocoa butter	0·00	0·00	0·00	0·00	18·88	3·59
Palm oil	0·00	0·00	16·52	3·15	16·52	3·15
Mineral mix S10026	9·48	0·00	11·65	0·00	11·65	0·00
Dicalcium phosphate	12·32	0·00	15·15	0·00	15·15	0·00
Calcium carbonate	5·21	0·00	6·41	0·00	6·41	0·00
Potassium citrate	15·64	0·00	19·23	0·00	19·23	0·00
Vitamin mix V10001	9·48	0·99	11·65	0·99	11·65	0·99
Choline bitartrate	1·90	0·00	2·33	0·00	2·33	0·00
FD&C Red Dye #40	0·01	0·00	0·00	0·00	0·06	0·00
FD&C Blue Dye #1	0·00	0·00	0·06	0·00	0·00	0·00
FD&C Yellow Dye #5	0·04	0·00	0·00	0·00	0·00	0·00
Total	1000	100	1000	100	1000	100

**Table 2.** Diet composition

	Control low-fat (CLF)		Control high-fat (CHF)		Enriched high-fat (EHF)	
Nutrients and energy	Diet%	kcal%	Diet%	kcal%	Diet%	kcal%
Protein	19·2	20·0	24·0	20·0	24·0	20·0
Carbohydrate	67·3	70·0	41·0	35·0	41·0	35·0
Fat	4·3	10·0	24·0	45·0	24·0	45·0
Cholesterol	0·00	0·00	0·03	0·00	0·06	0·00
kcal/g	3·9		4·7		4·7	
Fatty acid (FA)*	FA%	kcal%	FA%	kcal%	FA%	kcal%
C16 : 0 (palmitic acid)	16·0	1·6	21·0	9·4	21·6	9·7
C18 : 0 (stearic acid)	7·7	0·8	11·6	5·2	10·1	4·6
*cis*9–18 : 1 (oleic acid)	24·9	2·5	37·4	16·8	27·3	12·3
*trans*11–18 : 1 (*trans* vaccenic acid)	0·2	0·0	0·1	0·1	5·3	2·4
18 : 2*n*-6 (linoleic acid)	38·4	3·8	18·6	8·4	12·5	5·6
18 : 3*n*-3 (α-linolenic acid)	5·3	0·5	1·3	0·6	0·8	0·4
*cis*9,*trans*11–18 : 2 (rumenic acid)	0·1	0·0	0·1	0·0	2·0	0·9
∑TFA	0·9	0·1	0·9	0·4	14·3	6·4
∑SFA	25·6	2·6	34·7	15·8	36·2	16·3
∑MUFA	3·9	0·4	43·1	19·4	41·9	18·9
∑PUFA	44·7	4·5	21·3	9·6	20·3	9·1
*n*6-/*n*3-PUFA	7·1	–	12·3	–	13·4	–

TFA, *trans*-FA.

∑TFA: Sum of fatty acids that contain at least one *trans* double bond (*trans*-18 : 1+conjugated 18 : 2 + non-conjugated non-methylene-interrupted 18 : 2 + conjugated 18:3).

∑SFA: Sum of SFA.

∑MUFA: Sum of MUFA (*trans*16 : 1+*trans*18 : 1+*cis*16 : 1+*cis*18 : 1).

∑PUFA: Sum of PUFA (*n*6-PUFA + *n*3-PUFA+ conjugated 18 : 2+non-conjugated non-methylene-interrupted 18 : 2 + conjugated 18:3).

* Fatty acids are presented as % of total fatty acids (FA%) and as % of dietary energy (kcal%).

**Table 3.** Detailed fatty acid composition of diets (% of total fatty acids)

Fatty acid	Control low fat (CLF)	Control high fat (CHF)	Enriched high fat (EHF)
14 : 0	1·01	1·21	2·99
16 : 0	16·01	20·98	21·64
18 : 0	7·71	11·62	10·13
20 : 0	0·21	0·22	0·18
22 : 0	0·14	0·08	0·07
24 : 0	0·04	0·04	0·04
∑SFA	25·58	34·74	36·15
*c*7–16 : 1	0·15	0·18	0·12
*c*9–16 : 1	0·80	1·52	2·98
*c*11–16 : 1	0·03	0·02	0·21
*c*9–18 : 1	24·94	37·43	27·29
*c*11–18 : 1	1·54	2·23	1·03
*c*12–18 : 1	0·01	0·02	0·17
*c*13–18 : 1	0·06	0·09	0·31
∑*cis*MUFA	28·17	42·51	33·87
*t*6-*t*8–16 : 1	0·03	0·02	0·22
*t*9–16 : 1	0·03	0·01	0·10
*t*10–16 : 1	0·02	0·00	0·01
*T*11-*t*12–16 : 1	0·03	0·00	0·06
*t*14–16 : 1	0·01	0·01	0·02
*t*6-*t*8–18 : 1	0·03	0·02	0·32
*T*9–18 : 1	0·06	0·11	0·35
*t*10–18 : 1	0·06	0·09	0·33
*t*11–18 : 1	0·21	0·14	5·27
*t*12–18 : 1	0·02	0·02	0·32
*t*13-*t*14–18 : 1	0·10	0·06	0·69
*c*14-*t*16–18 : 1	0·03	0·02	0·20
∑*trans*MUFA	0·68	0·59	7·98
18 : 2*n*-6	38·41	18·61	12·48
18 : 3*n*-6	0·04	0·02	0·01
20 : 2*n*-6	0·24	0·31	0·02
20 : 3*n*-6	0·04	0·05	0·03
20 : 4*n*-6	0·10	0·14	0·03
∑*n*-6PUFA	38·85	19·22	12·57
18 : 3*n*-3	5·34	1·34	0·78
20 : 3*n*-3	0·05	0·08	0·03
20 : 4*n*-3	0·01	0·02	0·03
20 : 5*n*-3	0·02	0·02	0·03
22 : 3*n*-3	0·01	0·01	0·00
22 : 5*n*-3	0·04	0·06	0·06
22 : 6*n*-3	0·01	0·02	0·01
∑n3PUFA	5·47	1·56	0·94
*t*11,*t*15–18 : 2	0·01	0·02	0·33
*c*9,*t*13–18 : 2	0·03	0·03	0·46
*t*8,*c*13–18 : 2	0·01	0·01	0·13
*t*9,*c*12–18 : 2	0·06	0·05	0·10
*t*11,*c*15–18 : 2	0·03	0·03	2·03
*c*9,*c*15–18 : 2	0·06	0·10	0·11
*c*12,*c*15–18 : 2	0·01	0·01	0·04
∑AD	0·23	0·28	3·22
*c*9,*t*11–18 : 2	0·08	0·08	2·02
*t*11,*c*13–18 : 2	0·01	0·02	0·65
*t*11,*t*13–18 : 2	0·01	0·00	0·08
Other *t*,*t*-CLA	0·04	0·05	0·10
∑CLA	0·16	0·17	2·85
*c*9,*t*11,*t*15–18 : 3	0·01	0·00	0·26
*c*9,*t*11,*c*15–18 : 3	0·02	0·02	0·43
∑CLnA	0·02	0·02	0·69
∑TFA	0·93	0·97	14·25

*c* , *cis*; *t*, *trans*; AD, atypical dienes; TFA, *trans*-FA.

The TVA + RA-enriched beef tallow was sourced from the subcutaneous fat of steers fed a diet containing 75 % hay and 25 % flaxseed-based concentrate^(20)^. Briefly, the subcutaneous fat (i.e. back fat) was ground through a 6 mm plate (Butcher Boy meat grinder Model TCA22, Lasar Manufacturing Co.), vacuum packaged, frozen and held at –40°C until rendering. Prior to rendering, vacuum-packaged ground fat was melted and heated to 60°C in a water bath. The melted fat was strained through cheesecloth and added to an equal volume of water at 60°C. The fat–water mixture was then left to cool overnight at 2°C, and rendered beef fat was collected from the surface. The resulting fat was analysed for fatty acids and then sent to Research Diets, Inc. (Brunswick), to incorporate into a high-fat diet (Table 1).

Body weight and food intake were measured weekly throughout the study. Energy intake was calculated from food intake and energy density (kcal/g) of diets. At week 15, blood sampling (tail vein) was performed in fed and 12 h fasted animals to measure blood glucose and insulin levels. After 19 weeks of dietary treatments, mice were euthanised using cervical dislocation. Blood was collected from the abdominal aorta into EDTA anticoagulant tubes, and plasma was obtained after centrifugation at 1000 *g* for 15 min at 4°C. Plasma samples were stored at –80°C until used for analyses. Epididymal adipose tissue and liver were collected and weighed. Tissues were flash-frozen in liquid nitrogen and stored at –80°C until further analysis.

### Measurement of plasma metabolites

Glucose levels were measured with a glucometer (Easy Plus II, Home Aid Diagnostics Inc.) via tail vein blood. Insulin levels were determined by an ELISA (Ultra Sensitive Mouse Insulin ELISA Kit, Crystal Chem) according to the manufacturer’s instructions. Plasma TAG and total cholesterol esters were measured using Infinity^TM^ reagents (TR22421 and TR13421, Thermo Fisher Scientific). Plasma alanine transaminase (ALT) was analysed by the University of California, Davis (UC Davis) Comparative Pathology Laboratory.

### Insulin and glucose tolerance tests

A glucose tolerance test (GTT) and insulin tolerance test (ITT) were performed during week 16 and week 17, respectively. For ITT, mice were fasted for 4 h and then injected intraperitoneally with human insulin (1 U/kg body weight; Novolin-R, Novo Nordisk). Values were measured before injection and at 15, 30, 45, 60, 90 and 120 min post-injection. For GTT, overnight fasted mice were injected with d-glucose (1 g/kg body weight), and blood glucose was measured before injection and at 15, 30, 60 and 120 min post-injection. Glucose levels at indicated time points for ITT and GTT were measured from tail vein blood as described above.

### Liver TAG content and staining

Liver samples were homogenised, put into a 2:1 chloroform and methanol mix and stored at 4°C overnight. Next, 0·7 % NaCl was added to the mix and stored for another 24 h at 4°C. The aqueous upper phase was aspirated and discarded, and the bottom phase was removed and dried with nitrogen gas. The sample was reconstituted with 2-propanol, and TAG levels were quantified using Infinity^TM^ reagents (TR22421; Thermo Fisher Scientific). For histological analyses, 4 % paraformaldehyde-fixed liver samples were paraffin-embedded, sectioned and haematoxylin–eosin-stained by the UC Davis Comparative Pathology Laboratory.

### Quantitative real-time PCR

Frozen livers were homogenised and extracted using reagent TRIzol (Ambion) and RNeasy Plus Mini Kit (Qiagen), with the quantity and quality determined using a NanoDrop ND-2000 Spectrophotometer (Thermo Fisher Scientific Inc.). After that, cDNA was generated using a Maxima First Strand cDNA synthesis kit with the same total RNA amount for every sample (Thermo Scientific Inc.). Samples were mixed with PowerUp™ SYBR™ Green Master Mix (Thermo Fisher Scientific Inc.) and relevant primer pairs to determine the cycle threshold (Ct) by an Applied Biosystems MiniAmp Thermal Cycler (Thermo Fisher Scientific Inc.). TATA box-binding protein (*Tbp*) was used as the internal control gene because it has been shown to be a stably expressed housekeeping gene in the mouse liver^(21)^. Primer sequences for *Tbp* and target genes involved in inflammation including adhesion G protein-coupled receptor E1 (also known as *F4/80*), monocyte chemoattractant protein 1 (*Mcp-1*), interleukin-1 (*Il-1*) and cluster of differentiation 36 (*Cd36*) are listed in Table 4. The amplification efficiency for each primer pair was calculated from the slope of the standard curve generated with serial dilutions of a pooled cDNA sample using the formula (*E* = 10^(−1/slope)^). The amplification efficiencies were between 90 and 105 % for all primer pairs used in this study. Relative mRNA expression of target genes was calculated using the ΔCt method with *Tbp* as the internal control gene. Each 96-well quantitative PCR plate was set up to include reactions for the target gene and the control gene for each sample. Target gene Ct values were normalised to that of *Tbp* using 2^−ΔΔCt^ method^(22)^, and the results were expressed as fold change relative to control.

**Table 4.** Gene-specific forward and reverse primer sequences used for qPCR

Gene*	Forward primer (5’-3’)	Reverse primer (3’-5’)
*Tbp*	TGAGAGCTCTGGAATTGTAC	CTTATTCTCATGATGACTGCAG
*F4/80*	TGTGTCGTGCTGTTCAGAACC	AGGAATCCCGCAATGATGG
*Mcp-1*	GCTGGAGAGCTACAAGAGGATCA	ACAGACCTCTCTCTTGAGCTTGGT
*Il-1*	TGCAGCTGGAGAGTGTGG	TGCTTGTGAGGTGCTGATG
*Cd36*	GATGACGTGGCAAAGAACAG	TCCTCGGGGTCCTGAGTTAT

qPCR, quantitative PCR; *Tbp*, TATA box-binding protein; *F4/80*, adhesion G protein-coupled receptor E1 (also known as F4/80); *Mcp-1*, monocyte chemoattractant protein 1; *Cd36*, cluster of differentiation 36.

* The forward and reverse primer sequences were adapted from van der Heijden *et al*.^(37)^

### Fatty acid analysis

The fatty acid composition of the liver was determined using GC. Briefly, tissue samples were freeze-dried and direct-methylated using dual acid–base methylation with sodium methoxide followed by methanolic HCl^(23)^. *Cis*10–17 : 1 methyl ester (Nu-Chek Prep Inc.) was added as an internal standard prior to the methylating reagent. Fatty acid methyl esters were analysed by GC using a CP-Sil88 column (100 m, 25 μm ID, 0·2 μm film thickness) in a TRACE 1310 gas chromatograph (Thermo Fisher Scientific) equipped with a flame-ionisation detector (GC-FID, Thermo Fisher Scientific). Each sample was analysed twice by GC using a 175°C plateau temperature programme^(24)^. The fatty acid methyl ester were quantified using chromatographic peak area and internal standard-based calculations^(25)^.

### Data analysis

Data were analysed using the mixed model procedure of SAS (version 9.3; SAS Institute) with cage as a random effect and treatment as a fixed effect, and time as a repeated measure when applicable (i.e. for ITT and GTT data). Prior to analysis, data were checked for normality using the Anderson−Darling test, and all data were normally distributed. Differences between means were considered significant at *P* < 0·05 using the Tukey–Kramer multiple comparison test. Data are expressed as means ± standard deviation.

## Results

### Animal outcome

**Fig. 1.**
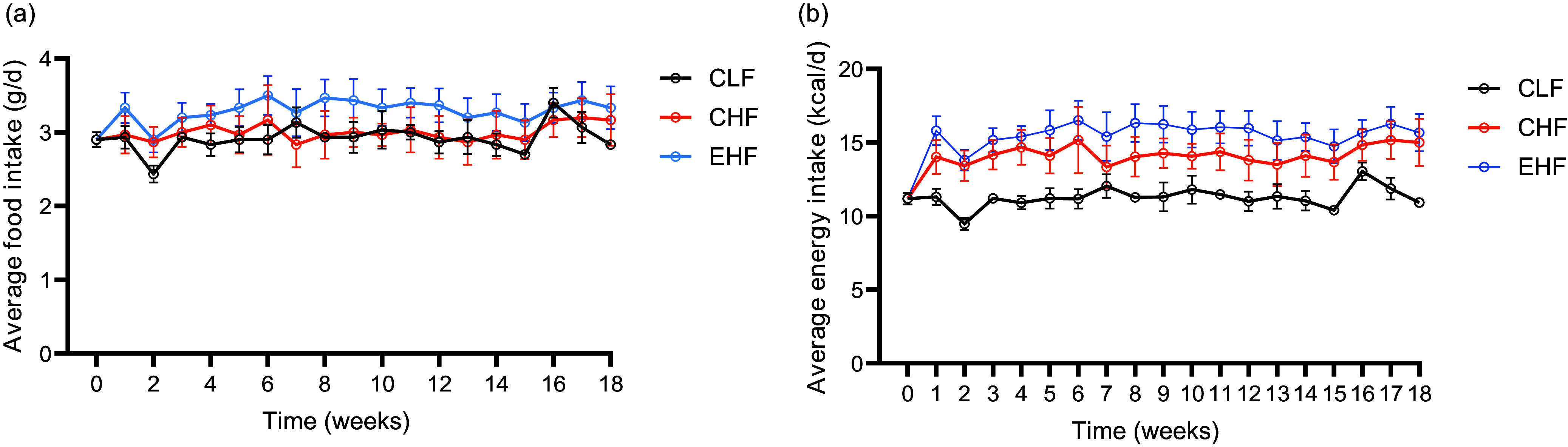
Food intake (a) and energy intake (b) of male C57BL/6J mice fed experimental diets: control low-fat diet (CLF, 10 % kcal from fat), control high-fat diet (CHF, 45 % kcal from fat) and high-fat diet with TVA and RA-enriched tallow (EHF, 45 % kcal from fat) for 19 weeks. Body weight (*n* 12/group) and energy intake (*n* 12/group) were measured weekly during the feeding period. Values are expressed as mean ± standard deviation. TVA, *trans* vaccenic acid; RA, rumenic acid.

The food intake during the experimental period was comparable among the treatments (Fig. 1(a)). As expected, the energy intake was significantly higher (*P* < 0·01) in both high-fat-fed groups (CHF and EHF) compared with the control low-fat diet group (Fig. 1(b)). Although the cumulative energy intake over the 19-week experiment was higher (*P* = 0·04) in EHF than in CHF, the body weight gain and final body weight were similar between the two groups (Table 5). Consistent with the increased body weight in high-fat-fed mice, adiposity was comparably increased, which was reflected in epididymal fat pad mass (Table 5). Both high-fat diets increased plasma levels of cholesterol compared with the control low-fat diet, whereas plasma TAG levels were not different among treatment groups (Table 5).

**Table 5.** Metabolic parameters

Parameter	Control low fat (CLF)		Control high fat (CHF)		Enriched high fat (EHF)		*P* value
	Mean	s d	Mean	sd	Mean	sd	
Body weight gain, g	8·03^a^	1·57	22·99^b^	3·12	24·49^b^	3·23	<0·001
Final body weight, g	32·64^a^	1·82	47·99^b^	3·54	49·34^b^	3·28	<0·001
Total food intake, g	358·8	15·35	361·64	27·85	404·36	20·79	0·08
Total energy intake, kcal	1381^a^	59·1	1711^b^	131·7	1913^c^	98·4	0·002
Fed insulin, pg/ml	1776^a^	1764	16546^b^	13 045	12882^b^	7739	0·004
Fasted insulin, pg/ml	381^a^	268	2428^b^	924·7	2363^b^	670	<0·001
Fed glucose, mg/dl	177^a^	16·8	230^b^	17·2	191^a^	21·1	<0·001
Fasted glucose, mg/dl	96^a^	13·5	153^b^	40·5	178^b^	27·9	<0·001
Epididymal fat pad, g	0·97^a^	0·36	1·94^b^	0·39	2·11^b^	0·3	<0·001
Epididymal fat pad,% of Body weight	2·94^a^	1·02	4·12^b^	1·17	4·29^b^	0·62	0·02
Plasma TAG, mg/ml	0·8	0·19	0·75	0·12	0·68	0·1	0·13
Plasma TC, mg/dl	73^a^	14·9	122^b^	28·1	120^b^	21·2	<0·001

CLF, control low fat (10 % kcal from fat); CHF, control high-fat diet (45 % kcal from fat); EHF, high-fat diet with TVA + RA enriched tallow (45 % kcal from fat).

Body weight gain, total food intake and total energy intake during 18 weeks on experimental diets.

Plasma glucose, insulin, total cholesterol (TC) and TAG concentrations on week 15.

Epididymal fat pad weight on week 19.

Values are expressed as mean ± standard deviation and are the average of twelve animals/group.

Means not sharing common letters (a–c) are significantly different (*P* < 0·05).

### Glucose metabolism

**Fig. 2.**
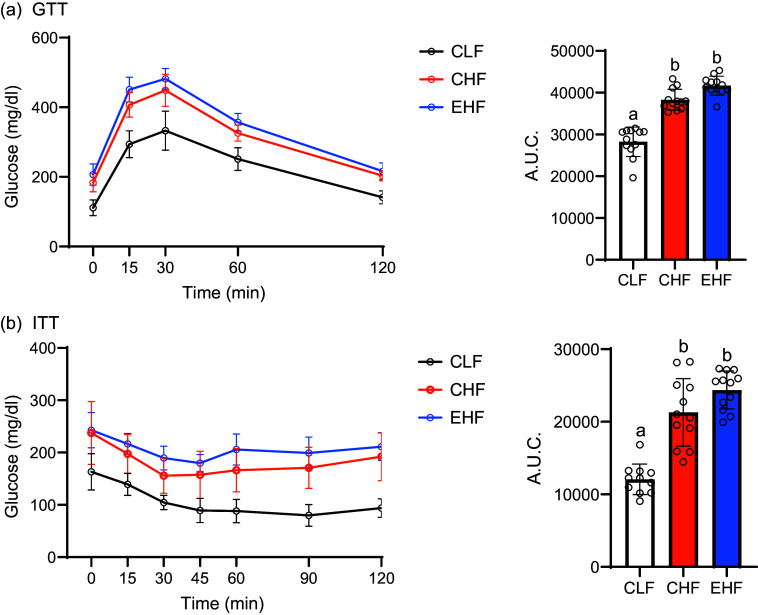
Glucose tolerance test (GTT) and its AUC at week 16 (a) and insulin tolerance test (ITT) and its AUC at week 17 (b) in male C57BL/6J mice fed experimental diets: control low-fat diet (CLF, 10 % kcal from fat), control high-fat diet (CHF, 45 % kcal from fat) and high-fat diet with TVA and RA-enriched tallow (EHF, 45% kcal from fat) for 19 weeks. Values are expressed as mean ± standard deviation and are the average of 12 animals/group. Values not sharing common letters (a) and (b) are significantly different (*P* < 0·05). TVA, *trans* vaccenic acid; RA, rumenic acid.

Consumption of high-fat diets (CHF and EHF) for 19 weeks resulted in increased fasted blood glucose and both fasted and fed insulin levels compared with CHF-fed mice (Table 5). However, the fed blood glucose levels were only significantly increased in the CHF group and not in EHF-fed mice (Table 5). Both high-fat diets induced glucose intolerance and decreased insulin sensitivity as evidenced by the GTT and ITT, respectively (Fig. 2(a) and (b)). The AUC for GTT and ITT was not statistically different (*P* > 0·05) between CHF and EHF groups (Fig. 2(a) and (b)).

### Liver fat content and inflammation

**Fig. 3.**
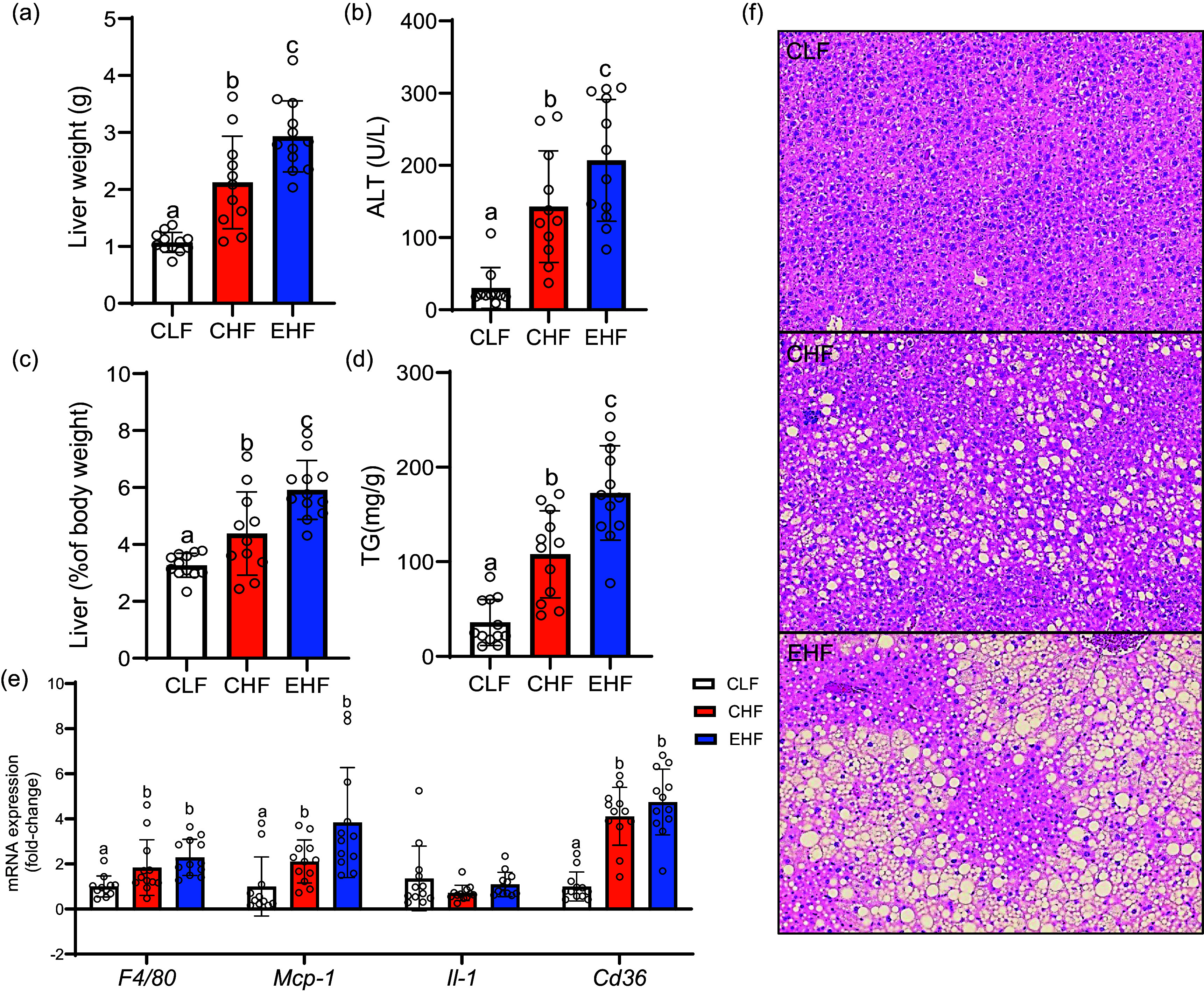
Liver weight in gram (a), alanine aminotransferase (ALT) concentration in plasma (b), liver weight as percentage of body weight (c), liver TAG content (d), mRNA expression of inflammation markers in the liver (e), haematoxylin and eosin (H&E)-stained liver sections (f) from mice fed different experimental diets. Male C57BL/6J mice fed experimental diets: control low-fat diet (CLF, 10 % kcal from fat), control high-fat diet (CHF, 45 % kcal from fat) and high-fat diet with TVA and RA-enriched tallow (45 % kcal from fat) for 19 weeks. Values are expressed as mean ± standard error and are the average of twelve animals/group. Values not sharing common letters (a)–(c) are significantly different (*P* < 0·05). TVA, *trans* vaccenic acid; RA, rumenic acid.

The effects of TVA + RA-enriched beef fat on hepatic steatosis and inflammation were assessed by measuring liver TAG content using a biochemical assay, inflammation markers by quantitative PCR and serum markers of hepatic inflammation/damage, including ALT (liver damage marker) and liver histology (Fig. 3). Both high-fat diets induced steatosis and inflammation as evidenced by liver TAG content (Fig. 3(d)) and histology (Fig. 3(f)) and mRNA expression of inflammation markers including *F4/80*, *Mcp-1* and *Cd36* (Fig. 3(e)). Furthermore, both the CHF and EHF groups had higher (*P* < 0·01) plasma ALT levels compared with the control group (Fig. 3(b)). When comparing the two high-fat-fed groups, EHF group had a higher (*P* < 0·05) liver weight (Fig. 3(a) and (c)), hepatic TAG content (Fig. 3(d)) and plasma ALT levels (Fig. 3(b)). However, there was no significant difference (*P* > 0·05) in hepatic expression of inflammation markers between EHF and CHF (Fig. 3(e)).

### Liver fatty acid composition

**Fig. 4.**
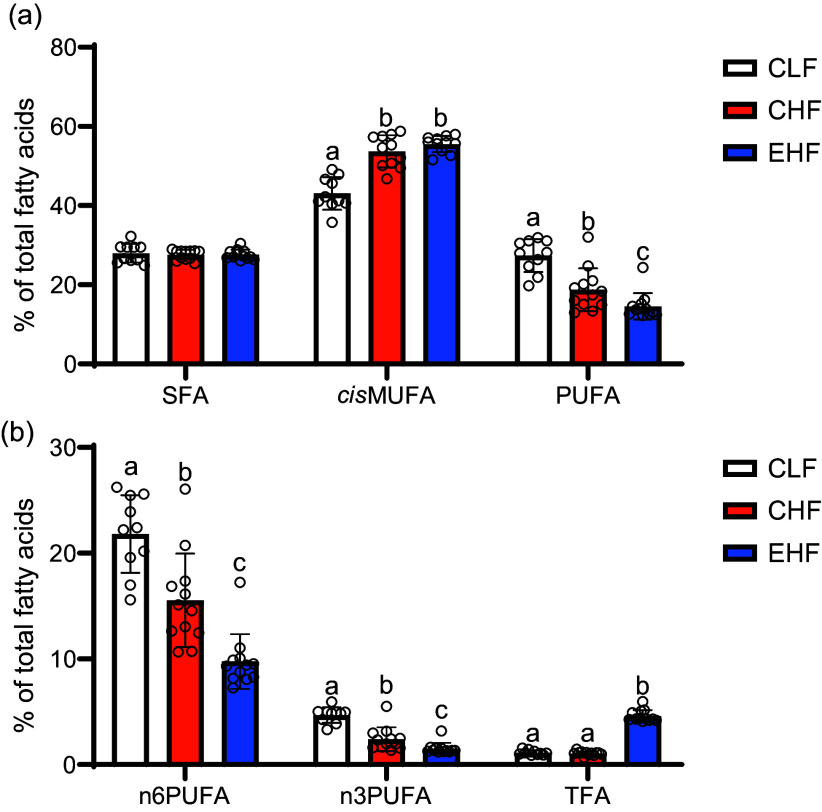
Hepatic content of (a) total SFA, total *cis*-MUFA, total PUFA and (b) total *n*-6 PUFA, total *n*-3 PUFA and total *trans*-FA in male C57BL/6J mice fed experimental diets: control low-fat diet (CLF, 10 % kcal from fat), control high-fat diet (CHF, 45 % kcal from fat) and high-fat diet with TVA and RA-enriched tallow (EHF, 45% kcal from fat) for 19 weeks. Data were analysed using the mixed model procedure of SAS. Values are expressed as mean ± standard deviation and are the average of twelve animals/group. Within each fatty acid type, bars not sharing common letters (a)–(c) are significantly different (*P* < 0·05). TVA, *trans* vaccenic acid; RA, rumenic acid.

Chronic consumption of high-fat diets (CHF and EHF) increased the hepatic concentrations of cis-MUFA at the expense of *n*-6 PUFA (Fig. 4(a) and (b)). Compared with the CHF group, the EHF group had a higher hepatic content of TFA isomers, including *t*9–16 : 1, *t*11–18 : 1 (TVA), *t*13–18 : 1, *t*14–18 : 1, *t*11,*c*15–18 : 2, *c*9,*t*11-CLA (RA), *t*11,*c*13-CLA and *c*9,*t*11,*c*15-conjugated linolenic acid (Table 6), and had a lower content of *n*-3 PUFA and *n*-6 PUFA (Fig. 4(b)) including α-linolenic acid (18 : 3*n*-3), docosahexaenoic acid (22 : 6*n*-3) and arachidonic acid (20 : 4*n*-6; Table 6).

**Table 6.** Fatty acid composition of liver (% of total fatty acids)

Fatty acid, % of total fatty acid	Control low fat (CLF)		Control high fat (CHF)		Enriched high fat (EHF)		*P* value
	Mean	sd	Mean	sd	Mean	sd	
14 : 0	0·50^a^	0·2	0·39^b^	0·05	0·48^a^	0·05	0·031
16 : 0	22·00^a^	1·4	23·72^b^	1·37	24·15^b^	0·77	0·005
18 : 0	5·01^a^	1·46	3·09^b^	0·86	2·36^b^	0·59	0·002
20 : 0	0·15	0·08	0·13	0·04	0·14	0·06	0·92
22 : 0	0·07^a^	0·03	0·03^b^	0·01	0·04^ab^	0·01	0·04
∑SFA	27·98	2·38	27·59	1·2	27·63	1·25	0·96
*c*7–16 : 1	0·91^a^	0·14	1·9^b^	0·36	2^b^	0·34	<0·001
*c*9–16 : 1	5·80^a^	1·17	3·11^b^	0·24	3·97^b^	0·46	<0·001
*c*11–16 : 1	0·04^a^	0·01	0·03^a^	0	0·15^b^	0·02	<0·001
*c*9–18 : 1	31·10^a^	3·14	42·24^b^	4·33	41·60^b^	3·34	<0·001
*c*11–18 : 1	4·23^a^	1·5	3·87^a^	0·85	4·81^a^	0·79	0·48
*c*12–18 : 1	0·00^a^	<0·001	0·00^a^	<0·001	0·06^b^	0·01	<0·001
*c*13–18 : 1	0·06^a^	0·03	0·04^b^	0·01	0·35^c^	0·03	<0·001
∑*cis*MUFA	43·07^a^	4·13	52·44^b^	5·84	54·43^b^	4·27	<0·001
*t*6-*t*8–16 : 1	0·00^a^	<0·001	0·00^a^	<0·001	0·04^b^	0·01	<0·001
*t*9–16 : 1	0·07^a^	0·01	0·05^a^	0·01	0·17^b^	0·05	<0·001
*t*10–16 : 1	0·00^a^	<0·001	0·00^a^	<0·001	0·02^b^	<0·001	<0·001
*t*11-*t*12–16 : 1	0·00^a^	<0·001	0·00^a^	<0·001	0·08^b^	0·01	<0·001
*t*14–16 : 1	0·02^a^	0·01	0·01^b^	<0·001	0·04^c^	0	<0·001
*t*6-*t*8–18 : 1	0·00^a^	<0·001	0·00^a^	<0·001	0·04^b^	0·01	<0·001
*t*9–18 : 1	0·06^a^	0·01	0·05^b^	0·01	0·08^c^	0·01	<0·001
*t*10–18 : 1	0·00^a^	<0·001	0·00^a^	<0·001	0·09^b^	0·09	<0·001
*t*11–18 : 1	0·08^a^	0·03	0·02^b^	0·01	0·55^c^	0·16	<0·001
*t*12–18 : 1	0·00^a^	<0·001	0·00^a^	<0·001	0·05^b^	0·01	<0·001
*t*13–18 : 1*	0·12^a^	0·04	0·16^ab^	0·03	0·18^b^	0·18	<0·001
*t*16–18 : 1	0·00^a^	<0·001	0·00^a^	<0·001	0·02^b^	0·02	<0·001
∑*trans*MUFA	0·35^a^	0·06	0·28^a^	0·04	1·41^b^	0·27	<0·001
18 : 2*n*-6	13·63^a^	4·04	10·41^ab^	2·76	6·92^b^	1·42	0·02
18 : 3*n*-6	0·47	0·37	0·26	0·14	0·1	0·03	0·23
20 : 2*n*-6	0·2	0·06	0·2	0·03	0·15	0·02	0·23
20 : 3*n*-6	0·82	0·44	0·69	0·14	0·46	0·14	0·24
C20 : 4*n*-6	6·45^a^	1·41	3·62^b^	1·61	1·94^c^	1·07	<0·001
∑*n*-6PUFA	21·82^a^	3·68	15·53^b^	4·43	9·75^c^	2·58	<0·001
18 : 3*n*-3	0·50^a^	0·23	0·20^b^	0·08	0·12^b^	0·04	0·02
20 : 3*n*-3	0·07	0·04	0·06	0·02	0·07	0·02	0·71
20 : 4*n*-3	0^a^	0	0·00^a^	0	0·03^b^	0·01	<0·001
20 : 5*n*-3	0·19^a^	0·06	0·08^b^	0·07	0·04^b^	0·02	<0·001
22 : 5*n*-3	0·27^a^	0·07	0·20^ab^	0·08	0·13^b^	0·06	0·02
22 : 6*n*-3	3·68^a^	0·6	1·87^b^	0·91	1·12^c^	0·44	<0·001
∑*n*3PUFA	4·70^a^	0·72	2·42^b^	1·13	1·52^c^	0·54	<0·001
*t*11,*t*15–18 : 2	0·00^a^	<0·001	0·00^a^	<0·001	0·04^b^	0·01	<0·0001
*c*9,*t*13–18 : 2*	0·00^a^	<0·001	0·00^a^	<0·001	0·23^b^	0·02	<0·001
*t*9,*c*12–18 : 2	0·00^a^	<0·001	0·00^a^	<0·001	0·02^b^	0·01	<0·001
*t*11,*c*15–18 : 2	0·18^a^	0·05	0·18^a^	0·04	0·62^b^	0·06	<0·001
*c*9,*c*15–18 : 2	0·03^a^	0·01	0·07^b^	0·01	0·11^c^	0·02	<0·001
*c*12,*c*15–18 : 2	0·00^a^	<0·001	0·00^a^	<0·001	0·01^b^	0·01	<0·001
∑AD	0·21^a^	0·06	0·25^a^	0·04	1·04^b^	0·07	<0·001
*c*9,*t*11–18 : 2*	0·06^a^	0·01	0·03^a^	0·01	1·44^b^	0·19	<0·001
*t*11,*c*13–18 : 2*	0·07^a^	0·02	0·07^a^	0·02	0·21^b^	0·02	<0·001
*t*11,*t*13–18 : 2	0·1	0·07	0·18	0·06	0·17	0·02	0·13
Other *t,t*-CLA	0·1	0·1	0·02	0·02	0·04	0·01	0·43
∑CLA	0·32^a^	0·06	0·31^a^	0·06	1·86^b^	0·18	<0·001
*c*9,*t*11,*t*15–18 : 3	0·02^a^	0·01	0·02^a^	0·01	0·09^b^	0·02	<0·001
*c*9,*t*11,*c*15–18 : 3	0·27	0·14	0·27	0·07	0·32	0·03	0·78
∑CLnA	0·29	0·15	0·29	0·07	0·41	0·05	0·38
∑TFA	1·13^a^	0·23	1·06^a^	0·19	4·6^b^	0·56	<0·001

*t*, *trans*; *c*, *cis*.

AD, atypical dienes; CLnA, conjugated linolenic acids; TFA, trans fatty acids.

*t13–18 : 1 co-elutes with *t*14–18 : 1; *c*9,t13–18 : 2 co-elutes with *t*8,*c*12–18 : 2; *c*9,*t*11-CLA co-elutes with *c*7,*t*9–18 : 2; *t*11,*c*13-CLA co-elutes with *c*9,*c*11-CLA.

Values are expressed as mean ± standard deviation and are the average of twelve animals/group. Means not sharing common letters (a–c) are significantly different (*P* < 0·05).

## Discussion

Previously, it has been demonstrated that short-term feeding (3–4 weeks) of pure TVA or TVA + RA-enriched beef fat can improve glucose homoeostasis and insulin sensitivity in JCR:LA-*cp* rats, a rodent model of dyslipidaemia and insulin resistance^(10,11)^. However, whether long-term consumption of these fatty acids can attenuate diet-induced glucose intolerance and insulin resistance is not clear. Thus, we conducted this study to evaluate whether long-term supplementation with a TVA + RA-enriched fat can attenuate high-fat diet-induced glucose intolerance and insulin resistance. We used high-fat diet (45 kcal% fat) fed male C57Bl6J mice, which is a reliable model for visceral obesity, glucose intolerance and insulin resistance. For the EHF diet, we used a tallow made from the subcutaneous fat of flaxseed-fed beef cattle, which is naturally enriched with TVA and RA^(20)^. It is noteworthy that TVA and RA (5·2 % and 2·02 % of total fatty acids, respectively; Table 3) were not the only TFA present in EHF. In fact, the EHF also contained other TFA, many of them with unknown health effects, including *trans* MUFA (e.g. *t*9–16 : 1, *t*13–18 : 1, *t*14–18 : 1), non-conjugated, non-methylene-interrupted dienes (*t*11,*c*15–18 : 2 and *t*11,*t*15–18 : 2) and conjugated linolenic acid isomers (*c*9,*t*11,*c*15–18 : 3 and *c*9,*t*11,*t*15–18 : 3). Among these, the level of *t*11,*c*15–18 : 2 was particularly high (2·02 % of total fatty acids, that is, the second dominant TFA in EHF). Thus, it is likely that the presence of other TFA such as *t*11,*c*15–18 : 2 in EHF may have influenced the outcomes related to glucose haemostasis and hepatic lipid accumulation in the present study.

### Both control and *trans* vaccenic acid + rumenic acid-enriched high-fat diet-induced obesity in mice

The increased body weight gain and adiposity in the high-fat treatments (EHF and CHF) were mainly due to the higher energy intake rather than the fatty acid composition of the diets. EHF and CHF feeding similarly increased body weight gain and epididymal fat pad weight compared with control low-fat diet. Consistent with our findings, feeding a high-fat diet containing butter enriched with TVA and RA to Wistar rats did not affect body weight or body composition compared with those fed a control high-fat diet^(9)^. Similarly, feeding pure TVA or RA had no effect on body weight and body fat accumulation in rodent models when compared with diets containing similar amounts of pure oleic acid or linoleic acid, respectively^(26,27)^.

### Beef fat enriched with *trans* vaccenic acid and rumenic acid did not alleviate glucose intolerance and insulin resistance caused by high-fat feeding in diet-induced obese mice

We found that long-term feeding of beef fat enriched with TVA and RA did not improve glucose homoeostasis in DIO mice, as evidenced by GTT and ITT data (Fig. 2(a) and (b)), and fasting blood glucose and insulin (Table 5). In contrast to our findings, feeding obese/insulin-resistant JCR:LA-cp rats a diet containing TVA + RA-enriched beef fat reduced fasting insulin and homeostatic model assessment for insulin resistance and reduced insulin secretion following a meal tolerance test^(11)^. In another study, feeding Wistar rats a high-fat diet containing TVA + RA-enriched butter ameliorated glucose intolerance compared with those fed regular butter^(19)^. The discrepancy between these studies and ours may be due to the differences in animal models, study duration and experimental diets.

### Beef fat enriched with *trans* vaccenic acid and rumenic acid resulted in increased hepatic fat accumulation in diet-induced obese mice

With regard to liver health, the EHF effects on the liver in the present study (increased liver weight and TAG content, inflammatory gene expression and elevated plasma ALT levels) resemble that previously reported for industrial TFA^(28–31)^. In rodents, dietary supplementation with partially hydrogenated vegetable oils, elaidic acid (*trans*9–18 : 1, the predominant *trans*18 : 1 isomer found in partially hydrogenated vegetable oils) or *t*10,*c*12-CLA has been shown to promote fat accumulation in the liver when compared with SFA or *cis*-unsaturated fatty acid^(32)^. Hepatic steatosis caused by partially hydrogenated vegetable oils has been mainly attributed to the upregulation of lipogenesis pathways in the liver, whereas *t*10,*c*12-CLA-induced hepatic steatosis is thought to be mainly caused by lipodystrophy or preferential fat accumulation in the liver at the expense of adipose tissues^(32,33)^. Furthermore, *t*10,*c*12-CLA-induced hepatic steatosis has been characterised by decreased hepatic content of long-chain PUFA in particular arachidonic acid (20 : 4*n*-6)^(33)^. Notably, the fatty acid analysis of the liver in the present study revealed a decrease in long-chain PUFA levels such as arachidonic acid and docosahexaenoic acid (22 : 6*n*-3) in the EHF-fed mice compared with that of CHF-fed mice. Nevertheless, the potential role of decreased long-chain PUFA levels in the induction and progression of steatosis needs further investigation.

It is noteworthy that short-term rodent studies with pure TVA and RA found either no effect or reduced liver fat content compared with oleic acid and linoleic acid, respectively^(10,26,34)^. Thus, it is possible that TFA, other than TVA and RA, contributed to the adverse effects of EHF on hepatic health in the present study. Moreover, given that the fat/oil sources used in CHF and EHF (Table 1) can contain variable amounts of other nutrients, such as cholesterol and fat-soluble vitamins, they might have confounded the treatment effects observed herein. For example, the EHF had higher cholesterol levels compared with CHF (0·06 *v*. 0·03 g/100g w/w; Table 2), which might have exacerbated hepatic lipid accumulation in high-fat-fed mice^(35,36)^.

### Conclusion

Contrary to our hypothesis, supplementation of TVA + RA-enriched beef fat did not improve glucose homoeostasis and worsened hepatic steatosis in high-fat-fed mice. However, the adverse effects on the liver could have been in part caused by other *trans*-FA present in the enriched beef fat, as well as other natural components such as cholesterol. Thus, more controlled feeding studies are needed to determine the health effects of TVA and RA and ruminant fats naturally enriched with these fatty acids.
